# Updates in Biliary Atresia: Aetiology, Diagnosis and Surgery

**DOI:** 10.3390/children12010095

**Published:** 2025-01-16

**Authors:** Mark Davenport

**Affiliations:** Department of Paediatric Surgery, Kings College Hospital, Denmark Hill, London SE5 9RS, UK; markday2@ntlworld.com; Tel.: +44-7711282653

**Keywords:** biliary atresia, Kasai portoenterostomy, BASM, adjuvant therapy, liver transplantation

## Abstract

Biliary atresia (BA) is an obliterative disease of the bile ducts affecting between 1 in 10,000–20,000 infants with a predominance in Asian countries. It is clinically heterogeneous with a number of distinct variants (e.g., isolated, Biliary Atresia Splenic Malformation syndrome, Cat-eye syndrome, cystic BA, and CMV-associated BA). Facts about its aetiology are hard to encounter but might include genetic, developmental, exposure to an environmental toxin, or perinatal virus infection. However, the cholestatic injury triggers an intrahepatic fibrotic process beginning at birth and culminating in cirrhosis some months later. Affected infants present with a triad of conjugated jaundice, pale stools, and dark urine and may have hepatosplenomegaly upon examination, with later ascites coincident with the onset of progressive liver disease. Rapid, efficient, and expeditious diagnosis is essential with the initial treatment being surgical, typically with an attempt to restore the bile flow (Kasai portoenterostomy (KPE)) or primary liver transplantation (<5%) if considered futile. Failure to restore bile drainage or the onset of complications such as recurrent cholangitis, treatment-resistant varices, ascites, hepatopulmonary syndrome, and occasionally malignant change are usually managed by secondary liver transplantation. This issue summarises recent advances in the disease and points a way to future improvements in its treatment.

## 1. Introduction

Biliary atresia (BA) can be defined as an obliterative condition of both the intra- and extrahepatic parts of the biliary tract almost inevitably present at the time of birth (see below for caveat). This leads to a stereotypical derangement in liver function with an inexorable increase in conjugated jaundice, pale stools because of absence of bile in the gastrointestinal tract and dark urine because of conjugated bilirubin spillage into the urine.

A feature of BA, distinguishing it from other neonatal cholestatic conditions (e.g., Alagille syndrome), is early progression of liver fibrosis ultimately leading to cirrhosis. It is this feature that really is the cause of much morbidity and for which we have no real solution.

BA is a rare disease with an incidence which varies across the globe. In the UK, Europe, and North America it is about 1 in 15–20,000 live births [1], though we have some recent data that suggests there may be hot spots in large cities such as Inner London with up to 1 in 10,000 live births [2]. Elsewhere in the world and specifically in East Asia, rates from 1 in 5–10,000 live births are common [3].

The aim of this report is to provide an up-to-date review of both the clinically relevant aspects of BA together with some framework on what we know of its possible origins and aetiology.

## 2. Do We Know What Causes Biliary Atresia?

**Figure 1:**
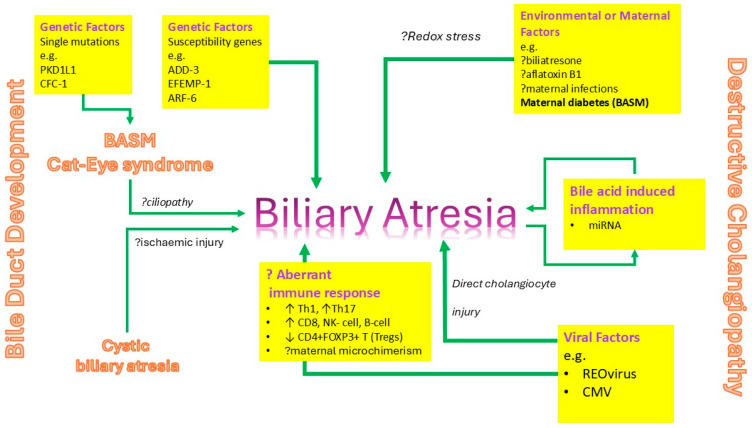
**Working hypothesis on the aetiological mechanisms underlying biliary atresia**. This implies that BA is a final common pathway with various independent mechanism—such as viral infection (±aberrant immune response), developmental derangement (±genetic mutation), ischaemic injury, environmental toxins etc.).

**Figure 2:**
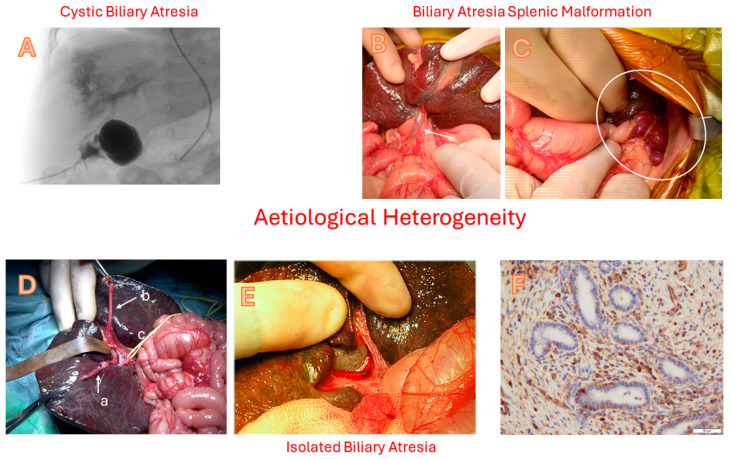
**Aetiological Heterogeneity: Cystic biliary atresia** with cholangiogram (part **A**) showing contrast retention in the cyst, absence of flow into the intestine, and abnormally fine, interconnecting etiolated intrahepatic bile ducts. **Biliary atresia splenic malformation syndrome** showing a pre-duodenal portal vein (part **B**, arrow) and left-sided polysplenia (part **C**, circled). **Isolated biliary atresia** (part **D**) showing (a) mobilized atrophic gallbladder, (b) umbilical vein and (c) sling around right hepatic artery. Cut surface of the porta hepatis before Roux loop reconstruction (part **E**). Photomicrograph of cut surface showing scattered biliary ductules within a fibroinflammatory stroma (vimentin-positive) (part **F**). Reprinted with permission from Ref. [5]. Copyright 2024 Springer Nature.

The short answer is—no we do not! However, there has been a lot of research into its origins, and we do have a working hypothesis, albeit a complicated one (Figure 1). This must explain the many variants of BA within the model and other clues research has provided (Figure 2). There may be many ways to arrive at the final common pathway of clinical BA. Some may be caused by derangements in the development of the biliary tree either early during the embryonic phase or much later within intrauterine life. For instance, there is some evidence, laboratory and clinical, to suggest that it may be a form of ciliopathy [4]. Other mechanisms might suggest the destruction and damage to an otherwise normally formed biliary tree [5]. Viral-induced damage during perinatal life of cholangiocytes allied to an aberrant, excessive pro-inflammatory response might better explain some observed cases.

### 2.1. Syndromic Biliary Atresia

BA, typically, is an isolated anomaly with no other associations. However, there is a group of syndromic anomalies which feature BA as a central feature. The first to be recognised is the **Biliary Atresia Splenic Malformation (BASM) syndrome** with splenic anomalies (typically polysplenia but also asplenia); situs inversus (30–40%); vascular anomalies (e.g., preduodenal portal vein (~40%), absence of the intrahepatic vena cava; cardiac anomalies (~50%) such as Tetralogy of Fallot and transposition of the great vessels; and malrotation [6,7]. The cardiac association is important and can determine the outcome depending on the level and degree of functional cardiac compromise. We coined the term **Cardiac Associated Biliary Atresia (CABA)** to emphasise that they do need special consideration [8].

A number of studies have tried to uncover a genetic basis for BASM, and some early reports identified single mutations in CFC1 (Cryptic, EGF-CFC Family Member 1) and NODAL (nodal growth differentiation factor) for instance most did not [8]. The largest genetic study involved exome screening of affected infants and their parent-identified mutations in the PKD1L1 (polycystin 1-like 1 gene) found on chromosome 16 but only in a relatively small proportion of about 10% [9].

We have also described the association of BA with the **Cat Eye syndrome** in 2008 [10]. This is much less common, and these infants had cardiac and ano-rectal anomalies as well as a characteristic genetic profile of aneuploidy of chromosome 22. They are named for the eye anomaly—coloboma.

### 2.2. Cystic Biliary Atresia

These infants have a cystic dilatation in an otherwise obliterated extrahepatic biliary tract and have a differential with cystic choledochal malformation. As such, they can be detected antenatally and more easily during the post-natal period using ultrasound. They make up 5–10% of most series and historically were the ones that were classified as “correctable” because of their suitability for standard biliary reconstruction [11].

These are the only variants to be able to show a cholangiogram of the intrahepatic ducts; although this can vary from a smooth tree-like appearance to a more disorganised primitive aggregation of irregular ductules, likened to that of a “cloud”. The cyst itself may be filled with clear mucus but in about 25% there is actual bile suggesting the persistence of communication with the intrahepatic ducts. These would be examples of the Type 1 BA of the Japanese classification.

### 2.3. CMV IgM +ve Biliary Atresia

The double-stranded DNA virus, cytomegalovirus (CMV) ubiquitously causes a number of diseases both before and after birth. In 1998, Bjorn Fischler et al. from Sweden showed CMV-IgM antibodies in serum from 38% of BA compared to 6% in age-matched controls [12]. This was a small series with an amazingly high proportion of affected infants. Association, of course, is not necessarily the causation but further evidence accumulated to suggest a stronger relationship. So, in London we identified our group of CMV IgM +ve infants who were older at the time of the Kasai portoenterostomy (KPE); with a higher degree of histological liver injury and fibrosis—even if age-corrected [13]. A later study also identified a different Th cellular profile than isolated BA, with a predominance of Th1 cells (vs. Th17 cells) [14]. Brindley et al. [15] from Denver, USA showed that just over half of BA infants had significant increases in γ-interferon-producing liver T cells in response to CMV exposure compared with a control group; suggesting that a higher proportion than might be expected were at least exposed to the virus. There are some demographic differences between CMV IgM +ve BA and those that are CMV -ve, and at least in England, there was a marked propensity for them to come from a non-Caucasian ethnic background.

For the first time, we also retrospectively identified how detrimental CMV was to outcome post-KPE with very few successful outcomes [13]. Later we were able to reverse this with active anti-viral therapy involving ganciclovir or valganciclovir [16].

## 3. Approaches to Diagnosis and Screening

Jaundice during the first two weeks of life could be said to be invariable in BA but about 10–20% of otherwise normal infants also have persisting jaundice beyond that period. The precise proportion is actually difficult to discern as only a few studies have been undertaken. One real-world study from Birmingham, UK found that 15% of normal infants at home were clinically jaundiced at 12–14 days, falling to 2.6% at 4 weeks, and 0.2% at 6 weeks [17]. Now, clearly the vast majority of these do not have BA, but the question remains how to identify that small proportion who do. Even if the search is restricted to those with conjugated hyperbilirubinaemia, biochemically inevitable in BA, it is still a minority diagnosis.

Clinically BA is difficult to diagnose. The patients will have pale, white stool and dark urine, but these have to be actively looked for, and later they have a degree of hepatosplenomegaly. Failure to thrive will be subtle until the disease is long-established. Some (<5%) will present with a coagulopathy and bleeding, uncommonly severe. This scenario is due to a vitamin K deficiency and will be more evident in those not availing themselves of routine Vitamin K prophylaxis at birth.

Biochemical derangement is inevitable with moderately high levels of the liver enzymes (aspartate aminotransferase and alanine aminotransferase (AST/ALT)), alkaline phosphatase (ALP) and more specific cholestatic enzyme γ-glutamyl transpeptidase (GGT). Increasing conjugated bilirubin defines and discriminates them from other pathological liver conditions but is not exclusive to BA. There has been much interest in a newer biochemical test based on matrix metalloproteinase-7 (MMP-7) levels. Nonetheless, this has not yet become a standard test because of the failure to define what constitutes an abnormal cut-off value due to the wide variation from a number of different manufacturers [18].

The differential diagnosis is long and illustrated in Table 1. It can be divided broadly into alternative surgical diseases such as choledochal malformation, inspissated bile syndrome, etc., and the alternative medical diseases such as Alagille’s syndrome, “idiopathic neonatal hepatitis”, Progressive Familial Cholestasis (PFIC) etc.

### Screening for Biliary Atresia

The first national screening programmes were developed in Taiwan and then in some Japanese prefectures and consisted of stool colour charts handed to mothers of newly born infants [19]. These were returned to be analysed centrally with those declaring colour anomalies invited back for further investigation. It seemed effective in Taiwan particularly in decreasing the time to KPE and reducing the proportion of late-attenders. However, it is still a subjective test with the onus on the parents to do something active. Mobile phone-based app technology (e.g., “PoopMD” and “PopòApp”) may add a degree of objectivity in the future [20,21] but much larger studies need to be performed before acceptance in any kind of screening programme.

A number of studies from the UK published in the 1990s showed that it was possible to measure certain components in the blood such as bile acids [22], and conjugated bilirubin [23] of newborn infants and for this to be a valid basis for population screening. More recently, this concept has been developed into an active screening programme in Texas based on the measurement of the conjugated fraction of bilirubin in whole blood obtained during the first few days of life. This is certainly sensitive to the diagnosis but not necessarily specific and a second-stage of investigation has been developed at about 2–3 weeks of age to improve specificity [24].

## 4. Surgical Management

Surgery has been the mainstay of treatment for BA since the 1920s and 1930s, although surgical success in that era was very limited and maybe only 5–10% of operated infants survived in the long-term.

In 1959, Kasai and Suzuki reported 12 infants operated on during the previous 8 years at Sendai in Japan [25]. These were usually regarded as “uncorrectable” by traditional techniques with inevitable failure. Surprisingly, some did show restoration of bile flow and long-term success using a more aggressive approach to the dissection within the porta hepatis, even if no actual bile ducts could be identified. A biliary reconstruction using a jejunal loop en Roux was settled upon and anastomosed to the transected porta hepatis. This became known as the Kasai portoenterostomy and its use spread slowly out of Japan becoming ubiquitous worldwide by the 1980s. It relies upon the presence of microscopic ductules in the porta which retain continuity with the intrahepatic bile ducts and which are therefore exposed with a transection of the otherwise solid biliary remnant in the porta hepatis.

Nowadays, the operation is still performed in >95% of cases and is mostly open though some centres in Japan and China controversially now advocate a laparoscopic approach [26].

For those that present late (defined at >100 days) then consideration for primary liver transplant may be given, though others have argued for its adoption in other more contentious scenarios [27]. More commonly, liver transplant is reserved for those who fail to normalise their bilirubin levels post-KPE or develop life-threatening complications such as recurrent cholangitis or treatment-resistant portal hypertension. In truth, over half of all children with BA will ultimately need this option.

### Adjuvant Therapy

Though the efficacy of the KPE operation is unquestionably based on anatomical there are things that could be adopted to improve the outcome following KPE. So, if there are no ductules uncovered by transection then no bile will flow and if the liver is so cirrhotic that the intrahepatic ducts have long vanished then no bile will flow.

About 50% of livers will show an inflammatory histological picture (not just those who are CMV +ve) with mononuclear cell infiltration, etc. This invites the possibility of immunomodulation, typically by administration of steroids. High-dose (e.g., >4 mg/Kg/day prednisolone tapered for 4–6 weeks) has been used by many centres [28,29]. Systematic analysis of the trial evidence has suggested a 10–15% improvement in initial outcomes, both jaundice clearance and native liver survival at 2 years [30].

For infants with positive evidence of CMV, then anti-viral therapy seems a logical addition. Our experience showed a dramatic improvement in clearance of jaundice and reduced need for liver transplantation [16], but we await evidence from other centres before accepting this as a standard part of management.

## 5. Outcomes and Complications

### 5.1. Portal Hypertension

BA has a definite propensity to progress at an early stage to fibrosis. This is different to other neonatal cholestatic conditions (such as α-1-antitrypsin deficiency and Alagille’s syndrome) and a key distinguishing feature.

There seem to be several mechanisms in the pathophysiology of this cholestatic liver fibrosis. One important feature is histological ductular reaction (DR) [31] which leads to the proliferation of reactive cholangiocytes and the transformation of formerly quiescent hepatic stellate cells to active myofibroblasts. α-Smooth muscle actin (α-SMA) is expressed characteristically and there is excess production of collagen [32,33]. Transforming growth factor (TGF-β1) and platelet-derived growth factor (PDGF) are seemingly major drivers of this process. Secretin and its receptor (SCTRA) normally regulate bicarbonate secretion allowing a protective “umbrella” protecting cholangiocytes from the effects of bile acids. This, also, has been suggested to play a role in the DR-fibrosis pathway [33]. The Helsinki group [34,35] showed that proliferating DR cholangiocytes overexpress SCTR mRNA at the time of KPE and that higher levels may be associated with reduced native liver survival. MMP-7, mentioned previously in the context of a diagnostic test, is an important contributor to the BA fibrotic process. This cleaves certain collagens and proteoglycan within the extracellular matrix and is an important component of the re-modelling process. Increased liver MMP-7 gene expression, localising to the biliary epithelium and periportal areas can be seen in BA extending several years into the post KPE-phase, even in those who have cleared their jaundice [35].

BA is characterised by the accumulation of bile acids in both liver and serum. There is a negative feedback on hepatic bile acid synthesis by the down-regulation of the rate-limiting enzyme, cholesterol 7α-hydroxylase (CYP7A1) via Farnesoid X-receptor (FXR) and small heterodimer partner (SHP). Bile acids also trigger increased production of FXR-mediated Fibroblast Growth Factor 19 (FGF19) in both the liver and small bowel. A recently published study from Helsinki and London demonstrated that high levels of serum FGF-19 at the time of KPE correlated with serum primary bile acids and, again, were associated with a reduction in subsequent native liver survival [36].

### 5.2. Cholangitis

A functioning KPE creates an effective bilio-enteric fistula and provides an opportunity for bacterial contamination of the intrahepatic bile ducts. Cholangitis never happens in those who never re-establish bile flow or clear their jaundice post-KPE. Nonetheless, it is common (up to 50% of some series), though probably over-diagnosed and if recurrent is the commonest reason for ultimate failure in those who have actually become anicteric [37].

The range of possible organisms is wide but derived from gastrointestinal flora and includes aerobic Gram-positive organisms, e.g., *Enterococcus faecium* and the Gram-negative organisms, e.g., *E. coli*, *Enterobacter cloacae* and *Klebsiella pneumoniae.* Anaerobic organisms or fungi rarely feature. Clinically, patients develop pyrexia, pale stools and worsening jaundice and should be treated urgently with intravenous broad-spectrum antibiotics (e.g., meropenem) for 1–2 weeks.

The efficacy of prophylactic antibiotics is questionable and there is no agreed consensus on the length of time or choice of antibiotic beyond KPE [38]. Recurrent cholangitis should prompt a search for bile lakes [39] (US and MR scan) due to undrained segmental biliary ducts and occasionally revision of surgical drainage may be indicated. In an older child, with otherwise exemplary liver function with a de novo cholangitis, a partially obstructed Roux loop may be to blame and again may be corrected surgically. In the absence of a surgical solution, then consideration should be given to the placement of an indwelling vascular catheter and long-term IV cyclical antibiotics in an attempt to break the cycle.

## 6. Controversies in the Management of Biliary Atresia

BA is potentially a lethal disease for which no surgeon from Morio Kasai onwards has reported complete surgical success with its usual treatment—the KPE. Arguments and opinions flourish in the absence of hard evidence but some deserve separate discussion.

### 6.1. Centralisation of Resources

**Figure 3:**
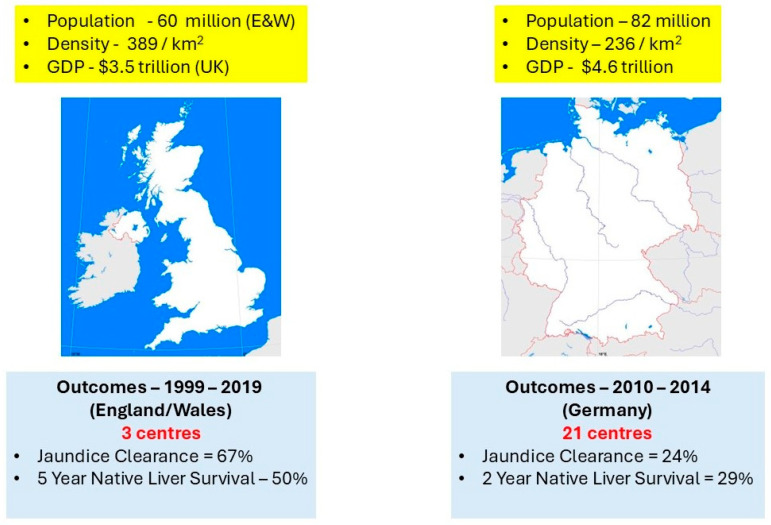
The centralisation effect: comparison between the biliary atresia management in England and Wales and Germany. [Background maps courtesy of https://alearningfamily.com/, accessed on 1 December 2024].

BA is a rare disease and KPE is an unusual operation not normally performed, in general, in paediatric surgical practice. Unsurprisingly, results can be poor in such circumstances. Germany is a first-rate nation with a high-quality health system. However, its hospitals are quasi-independent institutions that rarely refer to cases. The recently reported national outcomes with KPE are undeniably poor with an estimated clearance rate of ~25% [40]. Figure 3 illustrates the comparison between England and Wales where centralisation was made mandatory in 1999 and three centres anointed [41]. Subsequently, referrals increased from the remainder of the UK and for the past 10 years from the Republic of Ireland.

Centralisation has now been taken up by a number of Northern European countries as national strategy including Norway, the Netherlands, Sweden, and Finland with similar improvements in measured outcomes (e.g., [42]).

### 6.2. Adjuvant Therapy in Biliary Atresia

The role of steroids and antibiotics has been mentioned above, but other agents may have a role in improving the outcome [43]. Ursodeoxycholic acid is a secondary bile acid in humans with a long history of use as a synthetic compound in adult diseases, primary biliary cholangitis and sclerosing cholangitis. It may increase choleresis and alter bile acid proportions reducing some of the “toxic” components. There are also some in vitro benefits such as immunomodulation. However, actual evidence of benefit is rare in BA although its use has almost become ubiquitous in many centres.

Drugs of the ileal bile acid transporter inhibitor (IBAT) class such as maralixibat and odevixibat have been very successfully introduced in the treatment of PFIC and Alagille’s syndrome with a reduction in symptomatology if not disease progression [44]. As their name suggests (…ibat) their mechanism of action appears to be as an inhibitor of bile acid transport in the terminal ileum, preventing enterohepatic bile acid recirculation and an overall reduction in the endogenous bile salt pool. There are on-going trials in BA of both agents (e.g., EMBARK, maralixibat; BOLD, odevixibat) but recently reported results did not suggest a significant benefit, at least in the former [45].

There has also been some renewed interest in drugs designed to interfere with the fibrotic process within the liver itself. So, obeticholic acid is an agonist of the Farnesoid X receptor within the nucleus interfering with a number of inflammatory precursors and thereby inhibiting fibrosis. Again, it is the subject of a randomised trial, and the results might be expected in the next few years. Administration of polyunsaturated fatty acids (PUFA), specifically the n-3 PUFA, eicosapentaenoic acid (EPA) has also been investigated by several Japanese studies. This is believed to reduce the inflammatory process leading to fibrosis. In one non-randomised, prospective study of 57 infants (EPA *n* = 25), the clearance of jaundice rates and native liver survival to 2 years were similar but the EPA group had significantly lower platelet counts, and as a consequence lower APRi ratios at 2 years [46].

The promise of stem cell therapy, from whatever source, has been investigated in the context of BA. The most active institution promoting this is in Hanoi, Vietnam using bone marrow-derived mononuclear cells. One study in actual cirrhotic patients (*n* = 19) showed some improvement in biochemical values but very little evidence of change in the underlying fibrotic process [47]. A further study on 19 infants, involving umbilical vein infusion of such mononuclear cells at the time of KPE in 9, showed significantly improved biochemistry (most obviously in albumin and GGT levels) and PELD scores at 12 months and last follow-up. Nonetheless, the absolute clearance of jaundice rates in both groups was <50% throughout [48].

## 7. Conclusions

The management of the rare disease, biliary atresia, is the subject of a degree of scrutiny in many countries throughout the world. Although the results can appear capricious, unpredictable, and open to chance there is a discernible difference in some less than ideal results from otherwise respectable centres [49,50] and the obvious disparity with published results from larger or more experienced centres. There is renewed interest from the pharmaceutical industry in identifying newer adjuvant modalities which could improve the outcome and perhaps shift the balance to better retention of the native liver deferring liver transplantation to an older age group.

## Figures and Tables

**Table 1:** Differential Diagnosis of Conjugated Jaundice in the Newborn.

Medical			Surgical	
	Clinical Features	Genetics		Clinical Features
**“Idiopathic Neonatal hepatitis”**	Diagnosis of exclusion. Becoming less common.	n/a	**Cystic Choledochal malformation**	1 in 30,000 Antenatal US detected. Minority will have obstructive jaundice
**Parenteral nutrition**	↑↑ premature	n/a	**Inspissated bile syndrome**	↑ premature ↑ neonatal haemolysis ↑ neonatal sepsis
**Alagille syndrome**	1 in 30,000 Biliary hypoplasia Cardiac defects Eye-posterior embryotoxin “butterfly” vertebrae Facies	AD. *NOTCH2*, *JAG1* mutations	**Spontaneous perforation of bile duct**	Rare Bile ascites in a well child
**Alpha 1- antitrypsin deficiency**	M > F 1 in 2000 Variable presentation	AR *SERPINA1* mutations		
**PFIC**	↓ or normal GGT (except PFIC3) Variable presentation.	Canalicular transporter mutations (e.g., FIC1, *BSEP)*		
**Cystic fibrosis**	1 in 3000 Variable bile duct involvement.	AR *CFTR*		
**Neonatal sclerosing cholangitis**	<1 in 100,000	AR *DCDC2* *CLDN1*		
**Tyrosinaemia**	1 in 100,000 ↑↑ Quebec ↑ Finland	AR *FAH*		

AD, autosomal dominant; AR, autosomal recessive; PFIC, progressive familial intrahepatic cholestasis.
